# Cost-Efficient Video Synthesis and Evaluation for Development of Virtual 3D Endoscopy

**DOI:** 10.1109/JTEHM.2021.3132193

**Published:** 2021-12-01

**Authors:** Yaxuan Zhou, Rachel L. Eimen, Eric J. Seibel, Audrey K. Bowden

**Affiliations:** Department of Electrical and Computer EngineeringUniversity of Washington 7284 Seattle WA 98195 USA; Human Photonics LaboratoryDepartment of Mechanical EngineeringUniversity of Washington 7284 Seattle WA 98195 USA; Department of Biomedical EngineeringVanderbilt University 5718 Nashville TN 37232 USA; Department of Electrical Engineering and Computer ScienceVanderbilt University 5718 Nashville TN 37232 USA

**Keywords:** Virtual 3D endoscopy, 3D surface reconstruction, computer evaluation, medical simulation, video synthesis

## Abstract

Objective: 3D reconstruction of the shape and texture of hollow organs captured by endoscopy is important for the diagnosis and surveillance of early and recurrent cancers. Better evaluation of 3D reconstruction pipelines developed for such applications requires easy access to extensive datasets and associated ground truths, cost-efficient and scalable simulations of a range of possible clinical scenarios, and more reliable and insightful metrics to assess performance. Methods: We present a computer-aided simulation platform for cost-effective synthesis of monocular endoscope videos and corresponding ground truths that mimic a range of potential settings and situations one might encounter during acquisition of clinical endoscopy videos. Using cystoscopy of the bladder as model case, we generated an extensive dataset comprising several synthesized videos of a bladder phantom. We then introduce a novel evaluation procedure to reliably assess an individual 3D reconstruction pipeline or to compare different pipelines. Results: To illustrate the use of the proposed platform and evaluation procedure, we use the aforementioned dataset and ground truths to evaluate a proprietary 3D reconstruction pipeline (CYSTO3D) for bladder cystoscopy videos and compared it with a general-purpose 3D reconstruction pipeline (COLMAP). The evaluation results provide insight into the suggested clinical acquisition protocol and several potential areas for refinement of the pipeline to improve future performance. Conclusion: Our work proposes an endoscope video synthesis and reconstruction evaluation toolset and presents experimental results that illustrate usage of the toolset to efficiently assess performance and reveal possible problems of any given 3D reconstruction pipeline, to compare different pipelines, and to provide technically or clinically actionable insights.

## I. Introduction

Recent improvements in endoscopy have played a critical role in the early detection, monitoring and treatment of visceral cancers [1], [2]. Among them, virtual three-dimensional (3D) endoscopy has emerged as a promising technology for training and surgery [3]–[6], postoperative review and navigational mapping during robotic surgery [7], [8]. Conventional endoscopy suffers from the loss of spatial perception due to the projection of 3D structure into two-dimensional video frames. In contrast, 3D reconstruction pipelines for virtual endoscopy can produce 3D models of the shape and texture (visual pattern) of hollow organ cavities from monocular endoscope video frames that preserve spatial perception and are also easier to review, compare and annotate [9]–[12].

### A. Problem Statement

Determination of the clinical readiness of a given reconstruction pipeline requires objective evaluation tools that can assess its reliability and potential to work in a particular clinical scenario or to perform well under a variety of potential clinical scenarios. While 3D reconstruction pipelines have been developed for several clinical applications [10], [13]–[27], a robust set of evaluation tools has not been established. The lack of such tools makes it difficult to identify which aspects of a newly developed pipeline should be changed to improve its performance, or to compare different pipelines to determine which is better for a certain clinical application scenario.

### B. State of the Art

Evaluation of 3D reconstruction pipelines requires (1) a monocular endoscope video as input, (2) the 3D ground truth shape and texture of the organ to be reconstructed and (3) objective metrics to compare the reconstructed model and the ground truth. Importantly, the community should strive to use the same input datasets, ground truths and metrics for all pipelines to facilitate accurate and objective comparisons of newly developed pipelines.

While benchmarking datasets from the general-purpose 3D reconstruction community exist and can be used as video inputs for virtual endoscopy algorithms [28]–[32], their features do not resemble biological tissue nor do the movements and optical properties of commercial cameras mimic those of an endoscope. Hence, evaluations using these datasets do not generalize well to the clinical domain [32]. As a result, most virtual endoscopy developers perform evaluations using proprietary datasets [10], [13]–[27]. Not only are these datasets not broadly available, but they also represent only a limited range of clinical scenarios, which masks pipeline generalizability to different scenarios.

To obtain ground truth of organ shape, textures and camera poses, some prior works have used preoperational computed tomography (CT) scans of the organ or laser scans of physical phantoms and camera poses measured by commercial trackers [20], [22]–[25], [33]. However, these ground truths do not consider possible tissue deformation, the complexity of which is a major obstacle in the development of a robust 3D reconstruction pipeline for clinical use [27]. Moreover, scaling the size and variance of these datasets to permit evaluation over a range of clinical conditions (e.g., different settings for imaging speed, surface proximity, trajectory type, organ vascularity) is logistically challenging and costly. Computer simulation provides a solution for cost-effective generation of videos and ground truths having versatile properties [23], [34], [35]. However, most simulation systems for hollow organs like the colon, bronchus and abdominal cavity [36]–[39] were designed for virtual display during medical training and thus do not support data synthesis and evaluation for 3D reconstructions.

Finally, the metrics often used to evaluate reconstruction pipelines provide only a limited view of the pipeline’s performance [12], [20], [22]–[25], [33], making it hard to assess whether new pipelines are superior or inferior to existing options. For example, most works report subjective assessment of the 3D model’s visual appearance and/or the quantitative residual distance obtained after aligning the reconstructed 3D model with the ground truth model. The former practice is insufficient because it is qualitative and, therefore, unreliable. The latter practice only assesses accuracy of the reconstructed shape and can easily fail to correctly reflect the quality of the reconstructed model. For example, a reconstructed 3D model may be accurate (i.e., have a small residual distance) but incomplete, or the model may be accurate in shape while the reconstructed camera poses may be inaccurate, leading to inaccuracy of the final texture. Furthermore, neither practice reveals which are the problematic steps that restrict pipeline performance.

### C. Contributions

In this paper, we propose a new computer simulation tool (Section IIA) designed as a plug-in to Blender, a free and open-source 3D computer graphics software [40], for cost-efficient generation of synthetic benchmarking endoscope videos and associated ground truths mimicking a variety of clinical scenarios. Compared with similar Blender-based tools recently developed for generating simulated endoscopy videos [23], [40], our work demonstrates greater scalability to simulate a wider range of clinical scenarios, including tissue deformation. Moreover, the datasets generated with our tool allow for more robust evaluation of 3D reconstruction pipelines. To this end, we also propose a comprehensive set of metrics (Section IIC) that we suggest are necessary to reliably and correctly reflect the quality of reconstructed 3D models, reveal problematic steps in a given 3D reconstruction pipeline, and establish the working range of variables one might encounter in clinical use scenarios. The tools are publicly available in https://github.com/BBOL-team/bladderslam_EVS3D.git.

To demonstrate representative use cases for our tool, we use the simulation tool to generate an extensive benchmarking dataset (Section IIB) that is then used to evaluate CYSTO3D (Section IIIA–D), a proprietary 3D reconstruction pipeline described in a prior work for cystoscopy, which is endoscopy of the bladder. We show that the metrics we propose go beyond the traditional evaluation results to provide new insights that can help to guide future improvement of the pipeline or clinical protocol with which it will be used. The further step of comparing the performance of CYSTO3D and a general-purpose 3D reconstruction pipeline (COLMAP) (Section IIIE) reveals how our proposed tool and evaluation framework can guide selection of which pipeline is better suited for clinical translation. While the current paper focuses on bladder reconstruction from cystoscopy videos, our proposed tools are easily generalizable for other organs such as stomach.

There is currently no 3D reconstruction pipeline with technical readiness validated by preclinical or clinical studies, even though research in this field has been ongoing for over a decade [27]. We expect that the proposed tools can help standardize assessment of 3D reconstruction pipelines, thus accelerating their path to clinical translation to deploy virtual 3D endoscopy.

## II. Methods

### A. Endoscope Video Synthesis Platform: EVS-3D

We developed an endoscope video synthesis (EVS-3D) platform as a plugin within Blender 2.83 [40] using its python scripting application programming interface (API). EVS-3D simulates a virtual environment that comprises a virtual model for a hollow organ (phantom), a virtual camera to mimic the camera on the tip of the endoscope and a scan trajectory by which the camera captures images of the inner surface of the phantom. To create a synthesized video, the virtual camera moves along the trajectory, and images are rendered from the camera views as endoscope video frames. The synthesized video, the ground truth model associated with the virtual phantom used and the prescribed camera trajectory can be exported and used for evaluation of a reconstruction generated from the synthesized video.

**FIGURE 1.**
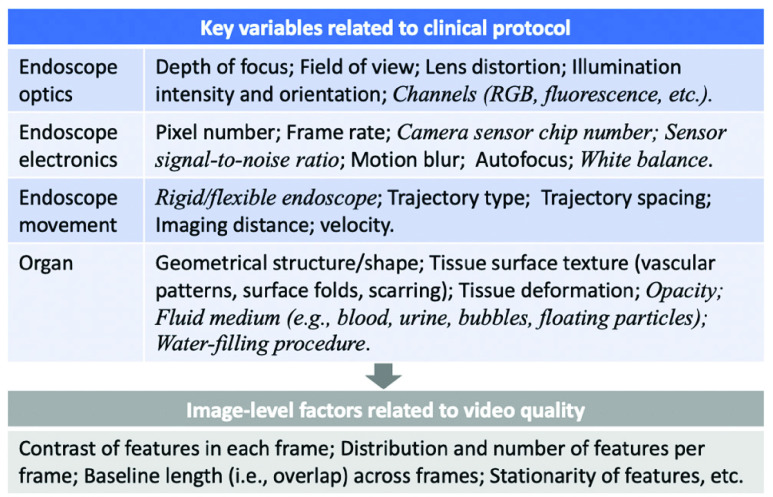
An inexhaustive list of key variables and image-level factors that influence the performance of 3D reconstruction pipelines.

EVS-3D enables the simulation of various clinical endoscopy scenarios in cost-effective manner. In particular, users can use the platform to generate multiple synthetic endoscopy videos by varying any of a number of user-adjustable key variables. These key variables represent differences in the clinical protocol that one might use to collect an endoscopy video; each variable has the potential to influence the quality of the acquired video and its subsequent reconstruction. **Fig. 1** shows an inexhaustive list of key variables (blue list in Fig. 1) – many of which can be adjusted in EVS-3D – that often intertwine to influence video quality, which is quantified by image-level factors (gray list in Fig. 1). For instance, field of view (FOV), frame rate, and endoscope trajectory (scan pattern) may influence the overlap across frames as well as the distribution of features per frame, both of which are crucial factors to determine whether the acquired video will be adequate for a reasonable reconstruction. Similarly, tissue deformation, which may arise from luminal wall expansion and muscle movements due to breathing, heartbeats and intervention during examination (e.g., urologists may push the belly to view larger regions in bladder), can change the stationarity of features on the object, making it difficult to perform accurate reconstruction with pipelines that are based on algorithms that assume rigidity of objects. While image-level factors directly indicate whether the video quality is sufficient for reconstruction, these factors are usually determined by key variables related to the clinical protocol. Thus, directly studying how key variables influence the final reconstruction is useful for providing actionable insights for clinicians and researchers developing reconstruction pipelines.

Among all the listed variables, there are, however, some variables that we choose not to simulate (italicized key variables in Fig. 1). For example, water-filling of the bladder is often conducted to obtain more working space during cystoscopy examination and would cause changes in the shape and texture when filled with different amounts of water. In such circumstances, evaluation of the reconstruction results becomes ill-defined, because the ground truth values of the shape and texture are changing. To enable a well-defined evaluation, we simplify the scenario and focus on whether a pipeline can reconstruct a 3D digital phantom (whose shape and texture are nearly fixed, having only small disturbances due to tissue deformation) from endoscope videos. In the clinical setting, we can satisfy the assumptions that the shape and texture of the organ do not change severely by making sure of the following: (1) the same amount of water is used to fill the bladder during different sessions; (2) frames acquired during water filling are discarded prior to the reconstruction.

**FIGURE 2.**
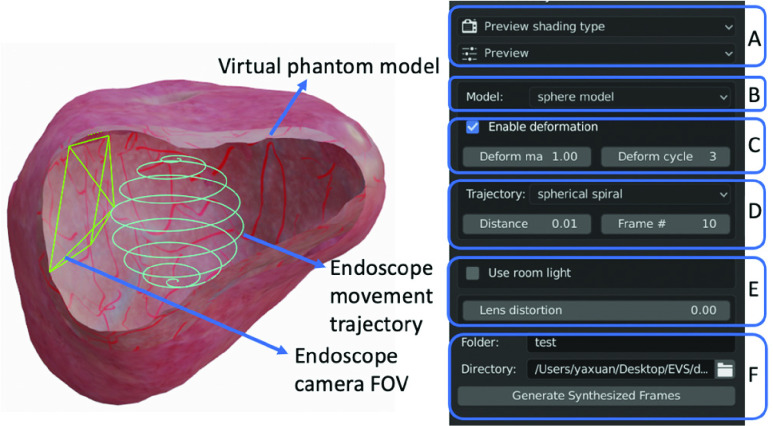
EVS-3D platform user interface. On the left is the Blender built-in 3D viewport showing a virtual phantom model and an endoscope movement trajectory (the cyan curves in the center of the model). The green frame indicates the endoscope camera FOV as a view frustum. On the right is a snapshot of the user panel for adjustment of (A) settings for the 3D viewport and some key variables during video synthesis, including (B) phantom model shape, (C) deformation, (D) endoscope movement related variables like trajectory type, (E) endoscope optics related variables like lens distortion, and (F) settings for file generation and exporting. Note that the user panel only shows the adjustment interface of a subset of the supported key variables. Other key variables are adjusted through Blender’s built-in interface.

**Fig. 2** shows the user interface of the EVS-3D platform, which displays the virtual objects in Blender’s built-in 3D viewport and packages adjustable key variables into the plug-in user panel. Following the taxonomy used in **Fig. 1**, we describe the key variables supported in EVS-3D platform.

With respect to optics in the virtual endoscope camera, EVS-3D supports adjustment of the depth of focus (DOF), FOV, lens distortion, illumination intensity and orientation. With respect to electronics in the virtual endoscope camera, EVS-3D supports adjustment of the pixel number, frame rate, sensor signal-to-noise ratio and motion blur. Users can set the above variables in Blender’s built-in object property interface.

**FIGURE 3.**
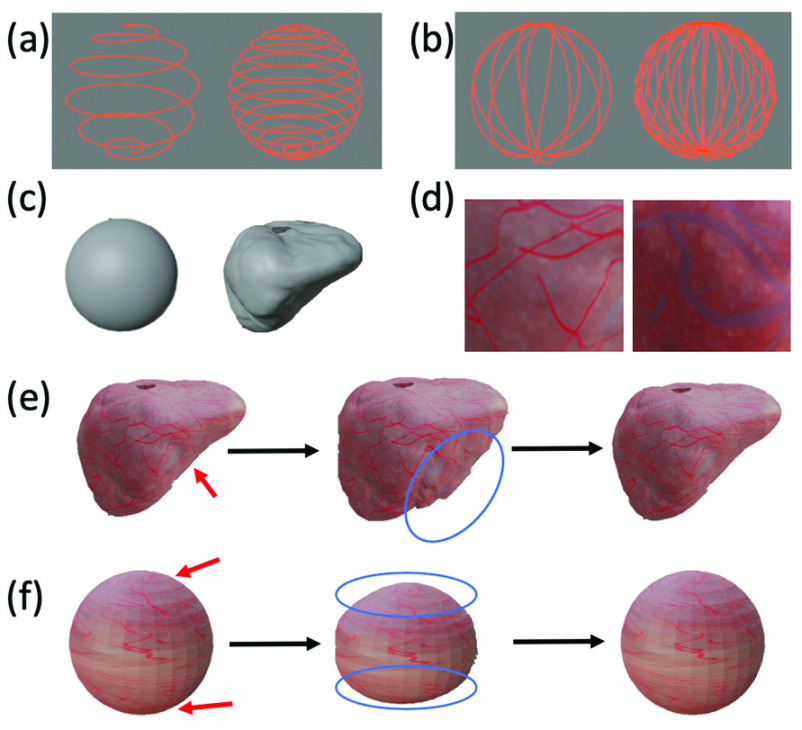
(a) Spiral trajectories with two different trajectory spacings. (b) Sine trajectories with two different trajectory spacings. (c) Preset phantom shapes: sphere, bladder. (d) Examples of cropped areas of synthesized bladder texture with varied contrast and feature density. Deformation cycle of (e) bladder-shaped and (f) sphere-shaped phantoms, both with synthesized bladder texture.

With respect to movement of the virtual endoscope camera, EVS-3D supports adjustment of the trajectory type, trajectory spacing (i.e., spacing between neighboring curves), imaging distance (i.e., distance between camera center to the inner surface of the virtual phantom model), camera velocity as well as customized trajectories. **Fig. 3** (a, b) shows examples of preset trajectory types (spiral, sine) and trajectory spacings. The user can create a customized trajectory by creating a curve-based object in Blender or by manually moving the mouse in the 3D view port to draw a trajectory curve. Jitter noise, simulating the imperfection of human movements, can also be added by manually moving the control points of any trajectory curve. With respect to the virtual phantom model, EVS-3D supports various phantom shapes, which allows users to evaluate the generality of a reconstruction pipeline on different organs. **Fig. 3** (c) shows two of the preset phantom shapes (sphere, bladder) currently available in EVS-3D. The preset organ shapes were extracted from CT scans of human participants; users can also add other shapes to represent other organs. The user can select preset phantom model from the user panel shown in Fig. 2 or create new phantom model by importing new 3D shape assets into Blender.

To set the texture of the virtual phantom model, the user can import high-resolution and high-contrast textures from wide-FOV endoscopic images and map the texture onto the 3D shape model in Blender’s UV Editing interface (Blender’s built-in interface for editing texture mapping on 3D model). If a high-quality texture is not available, EVS-3D also supports adjustment of the tissue surface texture through the synthesis of “vascularized” texture source images created by programmatically drawing vascular-like patterns on an either preset or user-defined low-resolution texture. Adjustable parameters include the maximum width and length of each vessel, the percentage of the texture containing vasculature and the color of the vasculature. **Fig. 3** (d) shows examples of synthesized bladder textures with different parameters. There are two gains of using texture with programmatically drawing vascular-like patterns. (1) For users that do not have access to high-quality real endoscopic textures, this feature provides an alternative to generate a customized bladder texture. (2) With programmatic drawing, one can generate different textures with various parameters (e.g., density of vascular patterns) and evaluate the influence of these parameters on 3D reconstruction performance. As real endoscopic textures usually have limited diversity, this evaluation would otherwise be hard to perform cost-effectively.

EVS-3D also supports the simulation of tissue deformation (e.g., to mimic heartbeats or intentional compression of the tissue during observation). One first creates a deformation profile by selecting a set of vertices on the phantom (indicated by red arrows in **Fig. 3** (e, f)) and by defining the maximum displacement (indicated by blue circles in **Fig. 3** (e, f)) and frequency of displacement (i.e., the number of deformation cycles in one second). **Fig. 3** (e, f) show snapshots of a complete deformation cycle, where the vertices (within the area marked by blue circles) move from an original location to a maximum displacement and then revert to their original locations. Users can choose from preset deformation profiles in the plug-in user panel or design their own as described previously. Once a deformation profile is selected, the user can set the displacement magnitude and displacement frequency by adjusting the “deform magnitude” and “deform cycle” parameters in the plug-in user panel.

### B. Generation of an Extensive Dataset

One possible use of EVS-3D is to modulate the aforementioned key variables over a range of values to generate an extensive dataset that can be used to assess a pipeline’s robustness/sensitivity over each variable. As the influences of different key variables on pipeline performance are usually entangled, the advantage of EVS-3D is that we can stringently control the key variables and isolate the one of interest without any extra cost.

**FIGURE 4.**
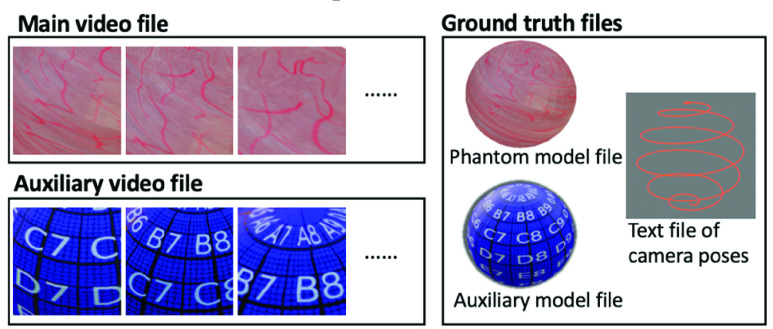
Each synthesis generates the following stored files: the main video file, the auxiliary video file and ground truth files (i.e., the phantom model file, the auxiliary model file and the text file containing the ground truth camera poses of all frames in the video).

We provide a representative extensive dataset synthesized using EVS-3D. For each synthesis, we first set the values of all aforementioned key variables. The virtual endoscope camera was then moved along the set trajectory to scan the complete inner surface of the virtual phantom model. All frames during the scan were exported and stored as the “main video.” Next, the same scan was repeated on a virtual auxiliary model that had the same shape as the phantom model but used a different texture (i.e., a multi-precision grid pattern with white coordinates on a blue background for best visual clarity). All frames during this scan were exported and stored as an “auxiliary video” (see Section IIC). The virtual phantom model, auxiliary model and the camera poses of all frames during the scan were exported to two model files and one text file as ground truths. Thus, each synthesis generates one main video file, one auxiliary video file and three ground truth files, as shown in **Fig.4**.

For the experiments described in this manuscript, we generated four groups (A-D) of several synthetic videos each by modulating over a subset of the key variables, as shown in **Table 1**. All synthesized videos in this dataset use a virtual phantom model with a spherical shape (diameter of 10 cm to mimic the distended bladder) and a synthesized bladder texture. We set key variables related to the virtual endoscope based on the specifications of a Karl Storz cystoscope (11272 VH/VHU), with simplifications: no lens distortion, sensor noise or motion blur.

**TABLE 1:** The Key Variable Settings Used for Our Extensive Dataset

	Group A	Group B	Group C	Group D
Trajectory type	spiral, sine	sine	sine	sine
Trajectory spacing (cm)	0.4	0.2, 0.3, 0.4,0.7	0.4	0.4
Imaging distance (cm)	4.0	4.0	2.0, 2.5, 3.5, 4.0	4.0
Tissue deformation (%)	0	0	0	0, 20, 60,100
Other key variables	Related to the virtual endoscope: DOF=50mm, FOV=120°, no lens distortion, fixed and even illumination, pixel number $=1920\times 1920$, frame rate=30Hz, no sensor noise, no motion blur, moving velocity=3cm/s. Related to the virtual phantom model: sphere shape, synthesized bladder texture.			

Group A contains two syntheses using different trajectory types. In this paper, we focus on two idealized trajectory types (i.e., no jitter) that are feasible in cystoscopy: (1) In the spiral trajectory (**Fig. 3** (a)), one continuously rotates the cystoscope shaft while simultaneously increasing the amount of shaft insertion, changing the bend of the tip when needed to scan the bladder in a spiral path. (2) In the sine trajectory (**Fig. 3** (b)), one continuously bends the cystoscope tip to scan vertically from the bladder dome to the bladder neck (entrance), rotates the cystoscope shaft by a small angle followed by another vertical scan, and then repeats the process until all 360-degrees have been covered. Note that the trajectory looks like a sine wave when flattened, hence the name.

Group B contains four syntheses with trajectory spacings of 0.2 cm, 0.3 cm, 0.4 cm and 0.7 cm. **Fig. 3** (b) shows a sine trajectory with a spacing of 0.7 cm on the left and one with a spacing of 0.2 cm on the right. Group C contains four syntheses with imaging distances of 2.0 cm, 2.5 cm, 3.5 cm and 4.0 cm.

Group D contains four syntheses with different levels of tissue deformation. We used the preset deformation profiles shown in **Fig. 3** (f) with a displacement frequency of 0.2 Hz (i.e., one deformation cycle takes five seconds). We define the deformation level to be the ratio of the actual maximum displacement during synthesis and the maximum displacement of the preset deformation profile. The deformation level can range from 0 (no deformation) to 100% (maximum displacement in the preset deformation profile).

### C. Evaluation Procedure

**FIGURE 5.**
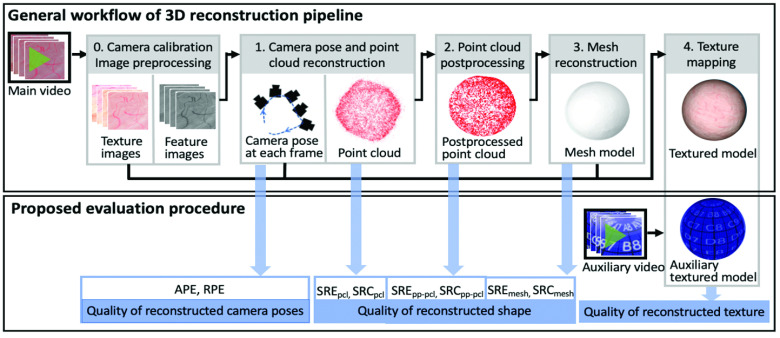
(Top) General workflow of a 3D reconstruction pipeline for a human organ from monocular endoscope video. (Bottom) Our proposed evaluation procedure and associated intermediate metrics to evaluate shape and texture.

Our proposed evaluation procedure is designed in accordance with the general workflow of 3D reconstruction pipelines for human organs from monocular endoscope video, as shown in **Fig. 5**. Such 3D reconstruction pipelines are typically composed of the following steps: (Step 0) Video frames are preprocessed to generate calibrated, feature-enhanced and texture-enhanced images. (Step 1) The camera pose at each frame and a 3D point cloud are reconstructed from feature images using algorithms like Structure from Motion (SfM) [41]. (Step 2) The reconstructed point cloud is postprocessed (e.g. filtering, smoothing) for noise reduction. (Step 3) A 3D mesh model is reconstructed from the postprocessed point cloud using algorithms like Poisson surface reconstruction [42]. (Step 4) A 3D textured model of the organ is generated by mapping texture images to the mesh model according to reconstructed camera poses of the mapped frames. Hence, a complete 3D reconstruction pipeline generates several intermediate outcomes (e.g., the reconstructed camera poses, point cloud, postprocessed point cloud, mesh model), and the final outcome is a textured model that captures both the shape and texture of the organ’s inner surface.

Since we consider emerging applications of virtual endoscopy such as training (i.e., identification of missing regions) and robotic guidance, we note the importance of evaluating the quality of both the shape and texture reconstruction produced by a given pipeline. However, most existing works perform either a qualitative evaluation or only report the accuracy of the reconstructed point cloud or mesh model, which only captures shape. These metrics fail to correctly reflect the quality of the shape and texture of the final product of the reconstruction; moreover, they do not assess intermediate steps of the pipeline and thus cannot reveal problematic steps responsible for poor final performance. For example, the quality of texture relies not only on the performance of step 4 but also the accuracy of camera poses recovered by step 1 and the quality of the mesh model reconstructed by step 3. The quality of the mesh model further depends on the performance of the steps 1 and 2. Steps 1-3 are designed to improve the quality of the reconstructed shape. Yet when these intermediate steps don’t perform well, the quality of shape may be degraded. Thus, it is also important to assess quality of the aforementioned intermediate outcomes.

To this end, we propose the following evaluation procedure associated with the steps described in **Fig. 5**:

(a) To evaluate outcomes of step 1, first assess the quality of the reconstructed camera poses via the absolute pose error (APE) and relative pose error (RPE). Then assess the quality (accuracy and completeness) of the reconstructed shape of the point cloud (pcl) via the shape reconstruction error (SRE $_{\mathrm {pcl}}$) and the shape reconstruction coverage (SRC $_{\mathrm {pcl}}$) metrics.
(b) To evaluate the outcome of step 2, assess the quality of the shape reconstruction on the postprocessed point cloud (pp-pcl) via SRE $_{\text {pp-pcl}}$ and SRC $_{\text {pp-pcl}}$.
(c) To evaluate the outcome of step 3, assess the quality of the reconstructed shape of the mesh model with the SRE_mesh_ and SRC_mesh_ metrics.
(d) To evaluate the final outcome of step 4, first repeat step 4 using auxiliary video frames as texture images to generate an auxiliary textured model. Then assess the quality of the textured model by visually inspecting it with respect to the ground truth auxiliary model. Note that step 4 does not change the reconstructed shape, so we do not need to assess the quality of the shape of the textured model.

Our proposed evaluation procedure uses three groups of metrics to assess the quality of reconstructed camera pose, shape and texture separately. These metrics are described below in more detail.

#### 1) Quality of the Reconstructed Camera Poses

Two metrics (APE and RPE) may be used together to quantify quality of the camera poses (i.e., how accurately the camera poses are reconstructed). First convert the recovered camera poses and ground truth camera poses to translation and rotation matrices in world coordinates. Then use scaling, translating and rotating transformations to align the two sets of poses. Finally, calculate the APE and RPE, defined in Eqns. (1, 2) [43], where $\text{P}_{\mathrm {i}}^{\mathrm {rec}}$ and $\text{P}_{\mathrm {i}}^{\mathrm {gt}}$ are, respectively, the reconstructed (rec) and ground truth (gt) camera pose of frame i. Note that matrix P can be a translation matrix, rotation matrix or a combination of both (the full camera pose). In this manuscript, APE and RPE are always calculated on the full camera pose, unless otherwise specified.

\begin{align*} {APE}_{i}=&\left \|{ {(P_{i}^{rec})}^{-1}{(P}_{i}^{gt})-I_{4\times 4} }\right \|_{F}, \\ APE=&\sqrt {\frac {1}{N}\sum \nolimits _{i=1}^{N} {APE}_{i}^{2}}\tag{1}\\ {RP}_{i,j}^{rec}=&{(P_{i}^{rec})}^{-1} {(P}_{j}^{rec}),\quad {RP}_{i,j}^{gt}= {(P_{i}^{gt})}^{-1}{(P}_{j}^{gt}) \\ {RPE}_{i,j}=&\left \|{ {(RP_{i,j}^{rec})}^{-1} {(RP}_{i,j}^{gt})-I_{4\times 4} }\right \|_{F}, \\ RPE=&\sqrt {\frac {1}{N}\sum \nolimits _{i,j}^{N} {RPE}_{i,j}^{2}}\tag{2}\end{align*}

Lower values of APE and RPE indicate higher accuracy of camera poses. APE focuses on the accuracy of the absolute pose while RPE focuses on the accuracy of relative poses (i.e., the relative pose between frame i and frame j) and thus should be less subject to accumulative drift. For example, a large APE and small RPE could indicate that a large error has occurred in the camera pose recovery for a particular frame that affects the APE of subsequent frames.

#### 2) Quality of the Reconstructed Shape

The quality of the reconstructed shape is related to both its accuracy and completeness. In particular, it is possible for a reconstruction to only cover a small portion of the intended shape, but with good accuracy (i.e., the model is incomplete), suggesting that accuracy alone is insufficient to evaluate the quality of the reconstructed shape. We use the SRE to quantify accuracy and the SRC to quantify completeness of the shape of a reconstructed model after Steps 1, 2 and 3.

First, normalize the size of the model bounding box over its longest edge and center the reconstructed model in MeshLab. Then use CloudCompare [44] to align the reconstructed model with the ground truth phantom model and perform iterative closest point (ICP) registration. Next, if the reconstructed or ground truth model is in mesh format, use Monte Carlo sampling in Meshlab to generate a set of randomly sampled vertices and export them as a new model in point cloud format. This is necessary since the SRE and SRC can only be calculated from models in point cloud format.

SRE is defined as the root mean squared (RMS) distance between all points in the reconstructed model and the ground truth, as shown in Eqn. (3), where $(x_{rec}^{i},y_{rec}^{i}, z_{rec}^{i})$ is the coordinate of vertex $v_{rec}^{i}$ in the reconstructed model, $(x_{gt}^{i}, y_{gt}^{i},z_{gt}^{i})$ is the coordinate of the ground truth vertex nearest to $v_{rec}^{i}$, and $N_{rec}$ is the total number of reconstructed vertices. Note that the range of this RMS distance is from 0 to 1.732 (the maximum length of diagonal in the normalized bounding box) and a lower value indicates higher accuracy of shape.

\begin{align*} SRE=\sqrt {\frac {\sum \nolimits _{i}^{N_{rec}} {{(x_{rec}^{i}- x_{gt}^{i})}^{2}+{(y_{rec}^{i}\!-\! y_{gt}^{i})}^{2}+{(z_{rec}^{i}-z_{gt}^{i})}^{2}} }{N_{rec}}} \\ {}\tag{3}\end{align*}

To calculate SRC, one can use the open source code from [32] to discretize the space into a grid of voxels whose edge length is defined by the user (we empirically chose 0.04 to provide a reasonable value for the SRC). All points of the model in point cloud format will then be binned into voxels in order to avoid the influence of point density on the metric. Defining an occupied voxel as “observed” when the distance to its closest ground truth voxel is below a specified threshold (we chose 0.01), the SRC can be calculated as shown in Eqn. (4): the ratio of the number of observed voxels over the total number of ground truth voxels. Note that the range of completeness is 0% – 100%, where 100% is the best case (i.e., all the surface area is fully covered by the reconstructed model).

\begin{equation*} SRC=\frac {number~of~observed ~voxels}{number~of~total~voxels ~in~ground~truth}\tag{4}\end{equation*}

We appreciate that the shape of an organ for a given endoscopy session may not be the same across all sessions. For example, how the bladder shape would change with intentionally applied force or different fluid filling conditions has not been well studied and thus is still an open question. In the scope of this paper, we make the following assumptions about the clinical context in which our proposed evaluation metrics are applied: the surgeons can control the amount of fluid filling and bladder distension to be about the same between different examinations so that the shape of bladder only exhibits differences in scale; and the video frames acquired during large, intentional application of force causing significant shape changes will be marked and discarded.

#### 3) Quality of the Reconstructed Texture

**FIGURE 6.**
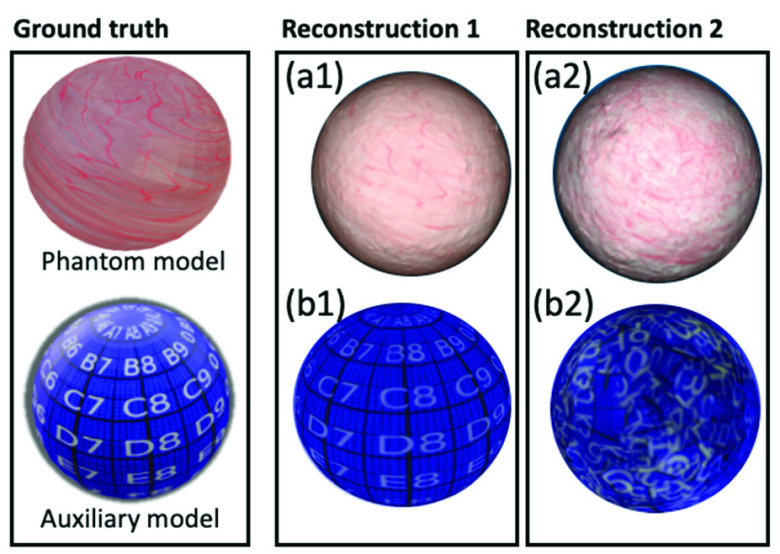
(Top row) The ground truth phantom model and textured models reconstructed from two synthesized videos in group B of our extensive dataset with trajectory spacings of 0.4 cm and 0.2 cm. (Bottom row) The ground truth auxiliary model and its reconstructed textured models from auxiliary videos, for evaluation of quality of reconstructed texture.

As the quality of reconstructed texture is hard to quantitatively evaluate, we propose to visually compare the ground truth model and the reconstructed textured model. In **Fig. 6**, we show the ground truth model on the left and two reconstructed textured models on the right. Reconstructions 1 and 2 are generated from two videos in group B of our extensive dataset with trajectory spacings of 0.4 cm and 0.2 cm, respectively. You can see that comparing the reconstructed textured models shown in **Fig. 6** (a1, a2) with the ground truth phantom model can be challenging due to the complexity of the texture.

Thus, we propose use of a multi-precision grid pattern with recognizable shapes (i.e., letters, numbers in white and grid lines in black) on a blue background. We wrapped the grid pattern onto the virtual phantom model and call the resulting model the “auxiliary model.” Then we used EVS-3D to render the auxiliary video during the video synthesis and used these views during the texture mapping step to generate the auxiliary textured model. If desired, one could potentially define multiple qualitative or quantitative levels using the multi-precision grid lines as reference.

## III. Results and Discussions

To illustrate use of the proposed EVS-3D platform, extensive dataset and evaluation procedure to evaluate a given 3D reconstruction pipeline, we performed reconstructions from videos in the extensive dataset (described in Section IIB) with two existing pipelines: CYSTO3D, a proprietary bladder 3D reconstruction pipeline [22] built upon several open-source backbone algorithms [42], [45]–[48], and COLMAP, a general-purpose 3D reconstruction pipeline [41], [49], [50]. In what follows we use the proposed evaluation procedure (described in Section IIC) to evaluate the quality of shape and texture reconstructions, to reveal problematic steps in CYSTO3D, assess its robustness over key variables (in Section IIIA – Section IIID) and to compare CYSTO3D and COLMAP (in Section IIIE). Clinically, the information gleaned from these types of evaluations can be used to guide the selection of key variables to be used during data acquisition. Technically, this information can identify target steps for algorithm refinement and guide selection of the optimal pipeline for a given clinical scenario.

### A. Influence of Trajectory Type for CYSTO3D

**FIGURE 7.**
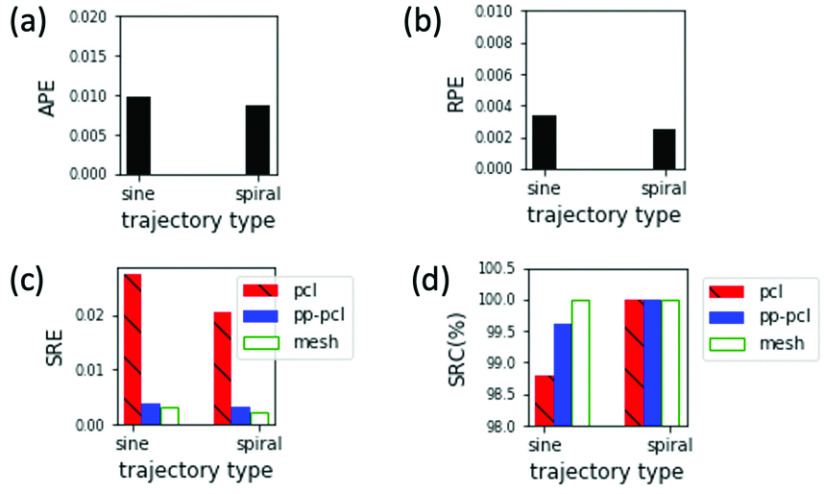
Evaluation results of reconstructions from group A videos in our extensive dataset. pcl: point cloud; pp-pcl: postprocessed point cloud; mesh: mesh model.

In a conventional cystoscopy session where clinicians manually operate the cystoscope, or in a tele-cystoscopy session where a robotic system moves the cystoscope with mechanical control, it is helpful to determine the planned trajectory for endoscope movement to ensure efficient and effective examination of the inner surface of the bladder. Our proposed EVS-3D platform and evaluation procedure can be used to quickly test out different trajectories. Here we use group A of our extensive dataset to evaluate CYSTO3D for the spiral and sine trajectories. The quantitative metrics calculated for the two scenarios are summarized in **Fig.7**.

In general, the spiral trajectory slightly outperforms the sine trajectory on almost all metrics. This indicates that a spiral trajectory is preferred for optimal robustness of the reconstruction pipeline. An interesting result is captured by **Fig.7** (d), which reveals that although the sine trajectory leads to lower SRC after step 1 of the pipeline, the SRC is comparable to that of the spiral trajectory after step 2 and step 3. This shows that when using the sine trajectory, the final reconstruction performance (especially completeness) will depend more on the performance of step 2 and step 3. Hence, if using the sine trajectory, the overall performance of the pipeline may be restricted by the performance of step 1 if steps 2 and 3 are inadequate to improve the quality of the reconstructed shape.

### B. Influence of Trajectory Spacing on CYSTO3D

The distance between neighboring curves of a trajectory (i.e., trajectory spacing) influences the overlap ratio between neighboring frames. We used group B in our extensive dataset to evaluate CYSTO3D over different trajectory spacings.

**FIGURE 8.**
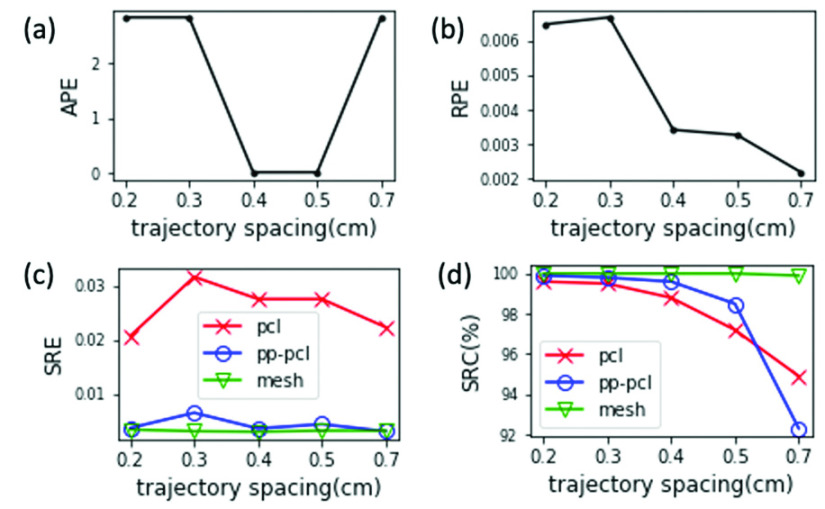
Evaluation results of reconstructions from group B videos synthesized with different trajectory spacings.

In **Fig. 8** (d), all SRC values monotonically decrease as the trajectory spacing increases from 0.2 cm to 0.7 cm. This may be because a narrower spacing likely leads to larger overlap between frames, which results in more feature points being detected, matched and reconstructed. In **Fig. 8** (c, d), SRE_mesh_ and SRC_mesh_, which indicate the accuracy and completeness of the final shape reconstruction, are comparable among all five spacings. Note that the quality of the reconstructed mesh model is better than that of the reconstructed point cloud model for all spacings as well. This suggests that steps 2 and 3 of the pipeline improve the quality of the shape from point cloud to mesh, as desired.

**Fig. 8** (a) reveals that the APEs of the full camera pose reconstruction for 0.2 cm, 0.3 cm and 0.7 cm are very large. For this experiment, we also calculated the APEs of the translation matrix and rotation matrices, as decomposed from the full camera pose. Interestingly, the APE of the rotation matrix is large while the APE of the translation matrix is nearly 0. These results clarify that the large camera pose error derives largely from an error from the rotation matrix, indicating a potential source of failure in the camera pose recovery part of step 1. **Fig. 6** (a2, b2) shows the reconstructed textured model from video acquired with a trajectory spacing of 0.2 cm. **Fig. 6** (a2, b2) reveals clear problems with the texture reconstruction, the deadly result of an inaccurate rotation matrix. This is a great example of using our proposed evaluation procedure to identify a problematic step (in this example, it is the recovery of rotation matrix of camera pose) within the reconstruction pipeline.

### C. Influence of Imaging Distance for CYSTO3D

The distance between the endoscope camera and the bladder surface being viewed (i.e., the imaging distance) strongly affects the quality of the acquired video. We empirically observed that, for a given frame rate and camera velocity, a larger imaging distance causes the vascular patterns to appear unfocused and blurred, decreasing the number of salient feature points, while too close of an imaging distance leads to reduced overlap between frames. Both extremes increase the difficulty of the feature-based matching process in the reconstruction pipeline, which is the key step to reconstruct the camera poses and the point cloud. The resolution of the vascular patterns and the degree of frame overlap are determined not only by the imaging distance but also by other key variables, including the camera FOV, frame rate, velocity, etc. Thus, an ideal imaging distance can only be selected once other factors are fixed, which is easy to test with the EVS-3D platform.

**FIGURE 9.**
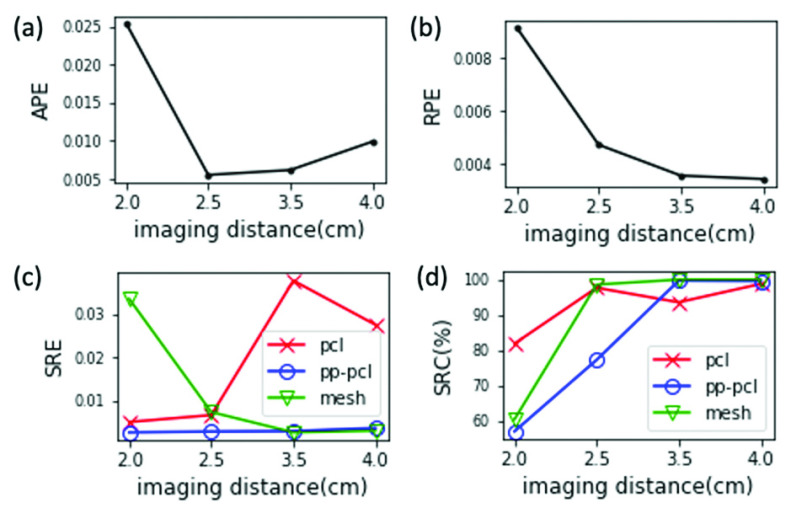
Evaluation results of reconstructions from group C videos synthesized with different imaging distances.

Here we used group C of our extensive dataset to evaluate CYSTO3D over different imaging distances. **Fig. 9** summarizes the evaluation metrics obtained. As the imaging distance increases from 2.0 cm to 2.5 cm, APE, RPE and SRE_mesh_ decrease (see **Fig. 9** (a-c)) while SRC_mesh_ increases (see **Fig. 9** (d)), indicating improved quality of both the reconstructed camera poses and shape. This shows that an imaging distance greater than or equal to 2.5cm may be preferred over a smaller distance for the pipeline to achieve a higher quality reconstruction.

We can further identify problematic steps within the pipeline by analyzing the metrics of each reconstruction. Taking the scenario with an imaging distance of 2.0cm as an example, we can see from **Fig. 9** (c), that SRE_pcl_ and SRE $_{\text {pp-pcl}}$ are reasonably good (i.e., small) compared to other distances tested, whereas SRE_mesh_ is large. This indicates the non-ideal performance of step 3, which negatively affects shape accuracy. Similarly, we can also see from **Fig. 9** (d) that step 1 already results in a moderate level of completeness of the point cloud model (SRC $_{\mathrm {pcl}}= 81$%), which further degrades after steps 2 and step 3 (SRC $_{\mathrm {mesh}}= 60$%). This indicates the non-ideal performance of steps 2 and step 3 on the completeness of the reconstructed shape. Thus, to improve the reconstruction performance, one either has to fine-tune the algorithm (especially step 2 and step 3) or change the imaging distance during clinical acquisition of endoscope videos.

As the imaging distance further increases from 2.5 cm to 4.0 cm, we can see from **Fig.9** (a, b) that APE increases while the RPE decreases. This may suggest that when the imaging distance gets too large, the reconstructed camera poses may incur a large error at some frame, which then accumulates in subsequent frames.

In **Fig. 9** (c, d), when the imaging distance increases from 2.5 cm to 3.5 cm, SRE_pcl_ increases and SRC_pcl_ decreases, indicating that accuracy and completeness worsen. Nonetheless, beyond 3.5 cm, SRE $_{\text {pp-pcl}}$, SRE $_{\mathrm {mesh}} < $ SRE_pcl_ and SRC $_{\text {pp-pcl}}$, SRC $_{\mathrm {mesh}} >$ SRC_pcl_, which indicate that shape quality (accuracy and completeness) is improved after step 2 and step 3. This may indicate that the negative effect of a slightly large imaging distance like 3.5 cm on step 1 can be mitigated by steps 2 and 3 if these steps are well-tuned at this particular setting. Actually, we can see that at an imaging distance of 2.0 cm, steps 2 and 3 worsen the shape quality (since SRE $_{\mathrm {pcl}} < $ SRE_mesh_ and SRC $_{\mathrm {pcl}} >$ SRC $_{\mathrm {mesh}}$), indicating that steps 2 and 3 are not well-tuned at this particular imaging distance. This shows that the performance of step 2 and step 3 is quite sensitive to the imaging distance. Thus, one would need to either pick an imaging distance where the pipeline works well, or improve the robustness of step 2 and step 3 if a larger range of imaging distance is required during clinical video acquisition.

### D. Influence of Tissue Deformation on CYSTO3D

Handling tissue deformation is a common challenge in 3D reconstruction of human organs. Since existing 3D reconstruction algorithms assume rigidity of the object, clinicians need to collect endoscope video frames with as minimal tissue deformation as possible during the endoscope procedure. Yet acquiring the perfect video without any deformation of shape and texture can be impractical. Even in the case of cystoscopy, where distending of the bladder during examination helps reduce deformation, there is still deformation caused by breathing, heart beats and occasional contact between the scope shaft and bladder wall. Thus, it would be helpful for clinicians to know the tolerance range on deformation that allows reasonable reconstruction performance so they can collect acceptable videos with reasonable effort. This information would also enable researchers tune the algorithm to handle the level of deformation expected with breathing, heartbeat artifacts or scope-organ contact.

**FIGURE 10.**
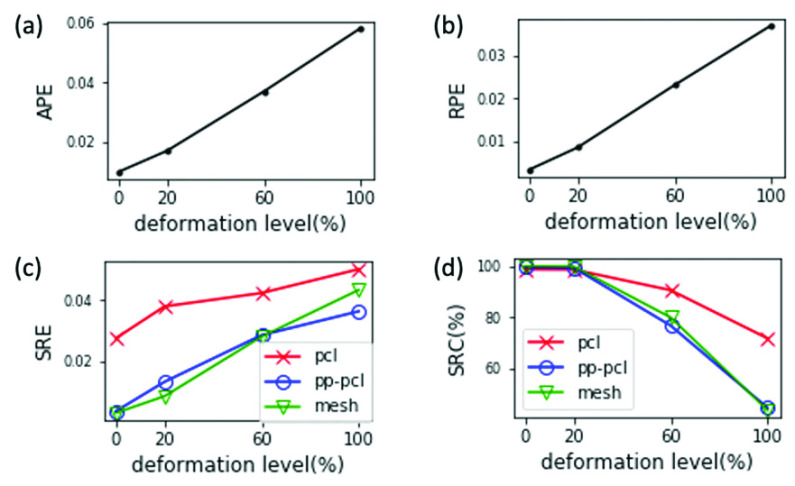
Evaluation results of reconstructions from group D videos synthesized with different deformation levels.

In **Fig. 10**, all the quantitative metrics monotonically degrade (i.e., APE, RPE and SRE increase, and SRC decreases) as the deformation level increases from 0% to 100%. This agrees with the expected trend: larger deformation in the video leads to worse quality of reconstruction. The evaluation statistics allow us to determine the upper bound of deformation that allows for reconstruction with a tolerable performance. For example, to achieve a completeness (SRC) of 90%, **Fig. 10** (d) shows that 20% of the preset deformation level is the maximum tolerable deformation able to guarantee the desired performance. Hence, if the deformation is large during the cystoscopy, clinicians may consider collecting more frames to ensure sufficient frames are collected with low deformation.

### E. Comparison of CYSTO3D and COLMAP Pipelines

**FIGURE 11.**
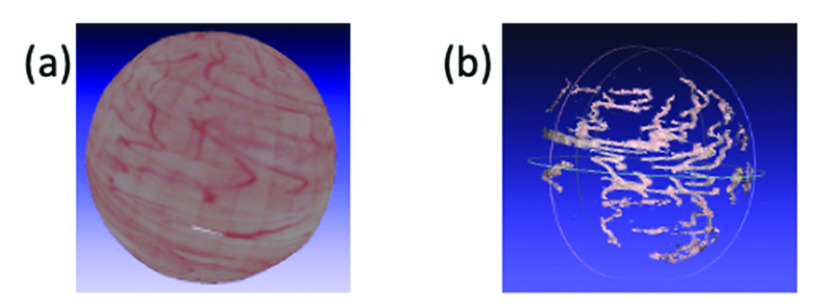
Visualization of reconstructed the textured model from (a) CYSTO3D and (b) COLMAP.

To compare two reconstruction pipelines, we used the synthesized data with spiral trajectory from group A of the extensive dataset. **Fig. 11** shows the final textured models reconstructed from CYSTO3D and COLMAP pipelines. The COLMAP pipeline performs poorly, largely due to the fact that it has not been fine-tuned to work well on bladder images. The reconstruction only captures areas containing vascular features and its evaluation metrics (APE = 0.00876, RPE = 0.00253, SRE $_{\mathrm {pcl}} = 0.0205$ and SRC $_{\mathrm {pcl}} =9.8$%) are significantly worse compared to those of CYSTO3D (APE = 0.00584, RPE = 0.00134, SRE $_{\mathrm {pcl}} = 0.0029$ and SRC $_{\mathrm {pcl}} =100$%). While the accuracy of those areas reconstructed by COLMAP is good, the completeness is very low. This specific result indicates that feature extraction in the point cloud recovery step of the COLMAP pipeline needs to be fine-tuned to reconstruct the shape with higher completeness.

## IV. Conclusion

In this paper, we proposed EVS-3D: a computer simulation platform for generating synthesized endoscope videos of the inner surface of human organs. EVS-3D can generate extensive datasets with corresponding ground truth information that can be used to evaluate and compare 3D reconstruction pipelines. We generated one such extensive dataset and also proposed an evaluation procedure to assess reconstruction pipelines. The evaluation procedure extends the types and range of metrics beyond those used in existing works. As such, it is able to comprehensively evaluate all intermediate and final outputs from the pipeline. Our evaluation strategy can better quantify the quality of the reconstruction of both shape and texture as well as assess pipeline robustness over a certain range of key variables during data collection, allowing it to reveal the source of problematic steps within a pipeline.

In this paper, we demonstrated the utility of these tools in the context of bladder cystoscopy and reported results on the evaluation of the bladder reconstruction pipeline CYSTO3D. We also used the extensive dataset and evaluation procedure to compare CYSTO3D with COLMAP, a general-purpose 3D reconstruction pipeline that has been used in stained stomach 3D reconstruction [25]. The primary goal of these experiments, results and discussion is to illustrate how researchers can utilize our tools to expedite algorithmic development and technology translation.

Potential directions for future work include developing better representations of trajectory curves to simulate more natural trajectories (e.g., the region-driven trajectory used by many clinicians), adding simulation of the mechanics of the endoscope shaft to better match the constraints of endoscope movement and improving the simulation of body fluids in the virtual phantom to better simulate artifacts from air bubbles and water flow.
